# Functional outcome after percutaneous screw fixation of acute scaphoid fracture

**DOI:** 10.6026/973206300214803

**Published:** 2025-12-15

**Authors:** Syed Hassan Raza Bokhari, Muhammad Tahir Iqbal, Muhammad Zarak Awais, Muhammad Almas Murad, Nouraiz shakoor, Mahtab zafar, Jawad Mustafa

**Affiliations:** 1 Department of Orthopaedics, Senior Registrar, Mayo Hospital, Lahore, Pakistan; 2 Department of Orthopaedics, Orthopaedic Trainee, Mayo Hospital Lahore, Pakistan; 3 Department of Orthopaedics, Orthopaedic trainee, Gulab Devi Hospital Lahore, Pakistan; 4 Department of Orthopaedics, Orthopaedic surgery Madina Teaching hospital, Faislabad, Pakistan; 5 Department of Orthopaedics, Rahbar Medical and dental college, Lahore, Pakistan; 6 Department of Orthopaedics, Shifa international hospital, Faisalabad, Pakistan

**Keywords:** scaphoid fracture, Herbert screw, functional outcome

## Abstract

Scaphoid fractures are common in adults and are prone to complications such as nonunion and avascular necrosis due to the bone's retrograde blood supply. Hence, this study evaluated the prevalence of excellent wrist functional outcomes following percutaneous Herbert screw fixation for acute scaphoid fractures in a tertiary care hospital. A total of 67 patients were assessed using the Mayo wrist score four weeks postoperatively. Most patients were young males with high postoperative functional scores (mean 94.48 ± 9.42), indicating favorable recovery. Percutaneous fixation with a Herbert screw proved to be an effective and reliable treatment option with excellent functional outcomes and minimal complications.

## Background:

Adult hand fractures involving the scaphoid bone are among the most frequent. Owing to its retrograde blood supply, the scaphoid's shape makes it particularly vulnerable to nonunion, avascular necrosis and other fracture-related complications [1]. Herbert screw fixation is a widely accepted treatment method for nonunion and displaced scaphoid fractures; however, the role of surgery in undisplaced or minimally displaced fractures remains controversial [2]. The current practice in our setup is conservative management using a scaphoid cast for patients with undisplaced or minimally displaced fractures, typically maintained for 8-12 weeks, which often results in wrist stiffness and significant loss of workdays [1, 2]. Excellent functional outcomes with conservative management of displaced or minimally displaced scaphoid fractures have been observed in only 72.0% of cases [3]. As surgical fixation offers higher union rates and allows earlier rehabilitation in displaced fractures, its use has gradually extended to less displaced injuries as well. Percutaneous screw fixation, in particular, has been associated with a high proportion of excellent results, with reported frequencies reaching 92.9% [4], 96.0% [5] and 88.5% [6]. These comparatively superior outcomes position percutaneous fixation as a potentially advantageous alternative to traditional conservative management. However, markedly lower success rates-approximately 40.0% [7], 40.0% [8] and 41.7% [9] have also been documented, reflecting significant variability in reported effectiveness. This inconsistency emphasizes the ongoing debate regarding the most appropriate management strategy and highlights the importance of further evaluation within local clinical contexts. A possible explanation for these discrepancies is the limited sample size of the studies (15-39 participants). Moreover, none of the studies [4, 5, 6, 7, 8-9] stratified outcomes based on important modifying factors such as occupation and duration of injury, which may significantly influence prognosis [10]. Additionally, no locally published data exist. Therefore, it is of interest to determine the frequency of excellent functional outcomes of the wrist following percutaneous screw fixation for acute scaphoid fractures, addressing the controversy in existing literature and the gap in local evidence.

## Materials and Methods:

The Orthopaedics & Spine Center conducted a retrospective analysis between March 2019 and August 2024. Approval from ethics was obtained. This study included sixty-seven patients who met the inclusion criteria and presented to the Orthopaedic outpatient and emergency department of Tertitary Care Hospital in Lahore. Using a 95% confidence level and a 9% margin of error, a sample size of 67 cases was determined. It was assumed that patients having percutaneous screw fixation for acute scaphoid fractures would have an excellent functional outcome of the wrist 93.3% of the time [11]. The data was gathered using a non-probability consecutive sampling technique. Patients with a local infection (either with or without discharge) exhibit redness and elevated local temperature in comparison to the contralateral side. According to the patient's history and clinical record, patients with pre-existing hand and wrist deformities may be congenital or acquired (such as burns, prior fractures, or surgical scars). Fractures that show up more than four weeks after trauma or that have a displacement of more than two millimeters in any plane are considered neglected. According to an x-ray of the affected wrist, patients with multiple fractures involving the distal radius, ulna, carpal, or metacarpal bones may also have impaired wrist function. Additionally, those with serious comorbid conditions that fall under the American Society of Anesthesiologists' (ASA) class ≥III were not allowed to participate in the study. Every patient gave a complete medical history and signed an informed consent form. The accompanying proforma will be used to notice and record the patient's age, gender, anatomical side, occupation and BMI duration since the injury. All of these patients had their scaphoid fractures fixed with percutaneous screws. The patient in Operation Theater was in a supine position without a tourniquet while under general anesthesia. A minimum 0.5 cm longitudinal incision was made at the most distal radial portion of the scaphoid while the wrist was in an ulnar deviation. Blunt dissection made the distal pole of the scaphoid visible. A 1.1 mm diameter percutaneous guide wire will be placed into the scaphotrapezial joint while being controlled by an image intensifier. The guide wire's length inside the scaphoid was measured using a depth gauge. The wire was taken out and a self-tapping headless Herbert screw was inserted utilizing intensifier control. Following the test dosage, a scaphoid cast will be placed post-operatively and each patient will receive a single intravenous injection of 1 g of ceftriaxone. In the evening of the procedure (daycare surgery), the patients were sent home on Tab. Panadol 2 Tab x TDS for 2 days and Tab. Augmentin 1gm BID for 5 days. At two weeks after the cast was taken off and rehabilitation began, the patients were seen outside. Four weeks following surgery, the Mayo score was used to evaluate the functional outcome and an outstanding functional outcome was recorded and noted as per the operational definition. To reduce bias, a single orthopaedic surgeon with at least 15 years of experience conducted all of the procedures and a single resident-the candidate himself-completed all of the postoperative functional assessments. Through exclusion, confounding variables have been managed. Age, BMI, Mayo Wrist Score and time since injury were all quantifiable and available as mean ±SD. The following categorical factors were computed by frequency and percentage: gender, anatomical side (right/left), occupation (sportsman, office worker, or laborer) and outstanding functional outcome of the wrist. To investigate effect modifiers, data were stratified by occupation, age, gender, BMI, duration since injury and anatomical side. Following stratification, a chi-square test of the association was used, with a p-value of less than 0.05 being considered significant.

## Results:

In this study, a total of 67 patients included diagnosed with scaphoid fracture; among these, the majority, 64(95.3%), were males while fewer, 3(4.5%) were female cases with an average age of 29.72 ± 11, ranged from (18-60) years. The average BMI of patients was 26.51± 3.22, ranging from (19.30-30.56) kg/m2. The mean duration of injury was 1.68 ± 0.76 weeks. The average MAYO score was 94.48± 9.42, ranging from (45-100). The majority of patients, 46(68.7%), were affected from the right side, while 21(31.3%) were affected from the left side. According to the occupation distribution, the results showed that more than half of the cases, 37(55.2%), were office workers (Table 1 - see PDF). Age significantly affected achieving the excellent outcome as most patients (85.7%) between 18-30 years had excellent functional outcomes compared to old patients between 31-60 years (57.1%). So there was a significant association found between age and excellent functional outcome. An insignificant association was observed between excellent functional outcome with gender, BMI, duration of disease, anatomical side and occupation as p>0.05 (Table 2 - see PDF).

## Discussion:

Scaphoid fractures are common and often difficult to diagnose and treat. Young people, who are most frequently affected, may lose employment and experience prolonged morbidity as a result of these injuries [12]. In our study, 32 (47.8%) patients were under 30 years of age, whereas 35 (52.2%) were above 30, indicating that young individuals frequently sustain scaphoid fractures. In another series, 13 (86.7%) patients were under 30, while only two (13.3%) were above 30, again suggesting a higher prevalence among younger adults [13]. Experience and knowledge of available treatment options are essential, even for initial management. When specific criteria are met, a favorable prognosis is achievable. To facilitate early wrist mobilization, Chen, Herbert, Fischer, McLaughlin and Maudsley recommended open reduction and internal fixation of acute scaphoid fractures using compression lag screws [14, 15]. The Herbert screw has been widely accepted as a treatment modality since it was first described by Herbert and Fischer in 1984 [16, 17]. Patients with acute displaced scaphoid waist fractures have been evaluated following open reduction and internal fixation using Herbert screws and K-wires through either volar or dorsal approaches. For acute undisplaced mid-waist scaphoid fractures, open reduction and internal fixation have been shown to be less costly from a societal perspective compared with cast immobilization and even when only direct costs are considered, internal fixation remains more economical than many widely accepted treatment techniques [18, 19]. In our study, four A2-type fracture cases were managed surgically, all showing complete healing with satisfactory clinical outcomes, compared with two patients who initially received cast treatment before eventually undergoing successful surgery. Both palmar and dorsal approaches are viable for Herbert screw insertion. The palmar approach preserves dorsal blood supply and effectively treats waist and distal pole fractures but disrupts volar carpal ligaments and provides limited proximal pole exposure. Conversely, the dorsal approach offers better access to the proximal pole but may compromise its delicate vascularity [20]. Fourteen of fifteen scaphoid fractures achieved successful fusion. Radiological union occurred at ten weeks postoperatively (range 8-16 weeks). Patients with delayed union during cast therapy demonstrated fracture union eight weeks after surgery. One patient with a proximal pole fracture achieved union at sixteen weeks. Another patient had no radiological union at the most recent follow-up (six months). Wrist flexion ranged from 35° to 75° (mean 40°-70°) and wrist extension averaged 60°. Based on the Modified Mayo Wrist Score (MMWS), the average range-of-motion score was 23.3 (10-25), average pain score was 21.3 (15-25), grip strength scored 24.6 (0-25) and activity level averaged 23.3 (15-25). Final follow-up grip strength averaged 4 (3-4.5). The average MMWS was 92 (45-100). Consequently, nine patients (60%) demonstrated excellent outcomes, five (33.3%) had good outcomes and one (6.7%) had a poor outcome. A representative case involved a 24-year-old male treated with open reduction and Herbert screw fixation for an acute mobile fracture. Radiographs at 14 months demonstrated complete union with excellent clinical recovery. No postoperative complications or malunions were observed. One patient with a proximal third fracture showed screw loosening, proximal fragment sclerosis and nonunion after missing follow-up for three months; although a vascularized muscle pedicle graft was planned, the patient declined further treatment. No patients exhibited wrist or scaphoid post-traumatic osteoarthritis at the latest follow-up (6-24 months). Scar tethering and hypersensitivity in four patients improved with physical therapy and scar massage; one patient with persistent scar sensitivity required a local steroid injection [10]. In another study, 60 cases of scaphoid nonunion were treated successfully using cancellous bone grafts with Herbert screw fixation. All patients were male and 50 cases involved the dominant hand. Of the total, 25 injuries resulted from road traffic accidents, 32 from falls on outstretched hands and three from assault. Ten patients had left-sided injuries, while 50 affected the right wrist. The average interval between injury and surgical fixation was approximately twelve months and the mean patient age ranged between 29.7 and 30.7 years. Occupational status included 13 clerical workers, 10 individuals with moderate wrist-loading jobs and 37 engaged in heavy wrist-loading occupations. All nonunion cases underwent routine postoperative follow-up and radiological evaluation [9].

## Conclusion:

Fixation with a Herbert screw for scaphoid fracture is a practical and effective treatment method with a good functional result and little risk of complications.
